# Characteristics of emergency department admissions with congestive heart failure in the United States: a Nationwide cross-sectional study

**DOI:** 10.1186/s12873-021-00564-7

**Published:** 2022-01-28

**Authors:** Xingyu Zhang, Peilin Qiu, Anna Prushinskaya, Yun Jiang, Hui Fan, Sheng Yang

**Affiliations:** 1 Department of Systems, Populations, and Leadership, University of Michigan School of Nursing, Ann Arbor, MI USA; 2 Thomas E. Starzl Transplantation Institute, University of Pittsburgh Medical Center, Pittsburgh, PA USA; 3 College of Literature, Science, and the Arts, University of Michigan, Ann Arbor, MI USA; 4 Department of Preventive Medicine, North Sichuan Medical College, Nanchong, China; 5 Department of Biostatistics, School of Public Health, Nanjing Medical University, Nanjing, China

**Keywords:** Congestive heart failure, Emergency medicine, National survey, Resource utilization

## Abstract

**Background:**

To understand the characteristics and clinical presentation of patients with Congestive Heart Failure (CHF) visiting the emergency department (ED), and to examine the factors associated with clinical outcomes and medical resource utilization amongst the studied population.

**Methods:**

We analyzed the 2014–2016 ED visit data collected by the National Hospital Ambulatory Medical Care Survey Emergency Department Subfile. We described patients’ characteristics and clinical outcomes after ED visits with CHF vs. without CHF. Logistic regression models were used to estimate the association between these characteristics and CHF.

**Results:**

ED visits with CHF visits represented 3.9% of annual ED visits (3,647,113 out of 92,899,685). ED patients with CHF were mostly non-Hispanic White (69.9%). Compared with other ED patients, those with CHF were older, including 71.2% that were were older than 60. ED patients with CHF were more likely to be admitted to the hospital (aOR: 2.56; 95% CI: 2.28–2.87) and intensive care unit (ICU) (aOR: 2.19; 95% CI: 1.77–2.71).

**Conclusions:**

This study describes the demographic, socioeconic, and clinical characteristics of patients who present to the ED with CHF through analysis of a comprehensive national survey. These patients require a higher level of emergency care due to their higher chance of admittance to the hospital and ICU.

**Supplementary Information:**

The online version contains supplementary material available at 10.1186/s12873-021-00564-7.

## Background

Congestive heart failure (CHF) is a national public health problem in the United States with significant prevalence and mortality [1, 2]. An estimated 5.7 million people in the United States have CHF [3], and CHF is one of the most common reasons for hospitalization among those 65 and older [4]. With an in increase in the aging population, the substantial healthcare utilization and cost burden of CHF is also growing [5, 6]. Estimated total health care expenditure attributable to heart failure, excluding cost related to comorbidities, is expected to be $160 billion [7].

While CHF is a complex clinical syndrome with a variety of etiologies and clinical presentations, many patients with CHF present with reduced left ventricular function and have the related symptoms of dyspnea with peripheral and pulmonary edema [8]. The absolute five-year mortality rate for CHF is estimated to be 50%, and epidemiological differences in incidence and mortality rates fall along age, racial, and gender lines [8]. For example, Black men represent the highest incidence of CHF, while White women represent the lowest. The highest prevalence of CHF is in those older than 65 [8]. Factors that influence prognosis include medication adherence; CHF patient medication adherence ranges from 7 to 90%, and medication nonadherence is thought to be a leading cause for CHF exacerbation [9]. Another factor associated with increased mortality is anemia, a common finding in patients with CHF that increases in incidence with worsening CHF [10]. Additionally, CHF patients who experience infection also experience increased mortality [11]. Finally, within the current context of the COVID-19 pandemic, CHF increases the risk of poor outcomes including intensive care mortality [11].

Visiting the Emergency Department (ED) can often be unavoidable for most patients with CHF. The ED is an important venue for care of patients with CHF [12, 13]. It was reported that the ED visits numbers for heart failure remained stable, from 914,739 in 2002 to 848,634 in 2010 (annual change − 0.7, 95% CI − 3.7 to + 2.5%) [14]. Limited previous studies have described characteristics of patients with CHF in the ED. In their secondary analysis of U.S [15]. ED visits for decompensated heart failure, Hugli et al. (2005) found that the average patient was 74 years old and that patients who were white were more likely to be hospitalized. Panduranga et al. (2016) described demographic and clinical characteristics of patients presenting with acute heart failure in Oman and concluded that the mean age was 63 ± 12, over half of the patients were male, and that the primary comorbidities were hypertension, coronary artery disease, and diabetes mellitus. Collins et al. (2016) described characteristics of patients with decompensated heart failure who were initially misdiagnosed as patients with non-decompensated heart failure and concluded that these patients were more likely to have a history of COPD and a lower b-type natriuretic peptide (BNP) level [16]. Further understanding the characteristics and clinical presentation of patients with CHF that visit the ED is an initial step towards improving their care and clinical outcomes [17–19]. A comprehensive understanding of ED utilization by patients with CHF may inform ways of reducing this population’s ED burden and ways of more effectively addressing these patients’ needs.

We performed a secondary data analysis on the National Hospital Ambulatory Medical Care Survey Emergency Department Subfile (NHAMCS-ED) to estimate the national characteristics of patients with congestive heart failure who utilize the ED, explore the association between congestive heart failure and related clinical presentations, and to follow health outcomes of patients with in the ED setting.

## Methods

The study design and statistical analysis is similar to our previous study on ED visits by patients with depression [20]. We performed a cross-sectional study of the 2014–2016 ED visit patient data collected by the National Hospital Ambulatory Medical Care Survey Emergency Department Subfile. The study included all adult patients records (age > 18; *N* = 42,832, Weighted *N* = 278,699,057) [21], a nationally representative, multistage, stratified probability sample of ED visits in the United States [22]. The survey design approach and database were introduced in the survey introduction documents [21, 22]. Generally, the NHAMCS-ED data is a yearly national survey of close to 300 hospital-based EDs from approximately 1900 geographic areas in all 50 states. A standardized data collection form was used to collect detailed information from around 100 patients per hospital-based ED. In total, the NHAMCS-ED included around 20,000–30,000 patients each year.

The primary study outcome is patient congestive heart failure status identified using the variable “congestive heart failure status,” which was checked from the patients’ electronic health records or claims data. NHAMCS defined congestive heart failure status as including “the inability of the heart to supply sufficient blood flow to meet the needs of the body. Does not include asystole or cardiac arrest.”

Secondary outcomes of the study included the Emergency Severity Index (ESI) (a five-level ED triage algorithm assigning patients a score from 1 [most urgent] to 5 [least urgent] on the basis of acuity and resource needs); hospital/intensive care unit (ICU) admission; blood test; medical imaging (including X-ray, CT, ultrasound, and MRI); clinical procedures (BiPAP/CPAP; bladder catheter; cast, splint, wrap; central line other; IV fluids; CPR; endotracheal intubation; incision & drainage; IV fluids; lumbar puncture; nebulizer therapy; pelvic exam; skin adhesives; suturing/staples; other); whether the patient left before triage or treatment; length of stay; and whether the patient died in the ED/hospital.

The study covariates that are examined include demographic characteristics (age, sex, race/ethnicity, region); socioeconomic status indicators, including residence (private home, nursing home, homeless, other) and insurance (private insurance, Medicare, Medicaid/CHIP, uninsured, other); day and mode of arrival; triage vital signs (temperature, pain scale, blood pressure, etc.); and reason for the ED visit.

### Statistical analysis

Population characteristics between congestive heart failure and non-congestive heart failure groups were described and compared. The proportion of medical imaging use among different racial/ethnic groups and covariates between groups were compared using chi-square tests. We used unweighted logistic regression to test the association between the outcome (ED patients with congestive heart failure) and the covariates. We imputed the missing with the median of each covariate when modelling the multivariable logistic regression. α = 0.05 was set as the statistical significance threshold. We used SAS (version 9.4) for analyses.

## Results

A total of 278,699,057 adult ED visits in the US were reported in the data, corresponding to approximately 92,899,685 annual ED visits from 2014 to 2016 (Table 1). Patients with CHF made up approximately 3.9% of these visits (equivalent to 10,941,339 or 3,647,113 annually). Basic characteristics are described in Table 1. Table 1 shows a comparison of demographic characteristics and disposition status of CHF-related and non-CHF-related visits. The mean age of the population is 47.2 (±19.6) (69.5 ± 15.6 for CHF and 46.2 ± 19.2 Non-CHF, *p* < 0.001). The proportion of the male patients is 43.0% (50.2% for CHF and 42.7% Non-CHF, *p* < 0.001).

**Table 1:** Baseline characteristics of patients presenting to the ED, stratified by congestive heart failure, NHAMCS 2014–2016

	Unweighted Sample, N (%)			Weighted Sample, N (%)		
	All	No Congestive heart failure	Congestive heart failure	All	No Congestive heart failure	Congestive heart failure
	42,832	41,106 (96.0)	1726 (4.0)	278,699,056	267,757,717 (96.1)	10,941,339 (3.9)
Male	18,469 (43.1)	17,618 (42.9)	851 (49.3)	119,751,766 (43.0)	114,262,696 (42.7)	5,489,071 (50.2)
Age, y						
18–39	17,912 (41.8)	17,835 (43.4)	77 (4.5)	118,068,691 (42.4)	117,591,074 (43.9)	477,617 (4.4)
40–49	6662 (15.6)	6559 (16.0)	103 (6.0)	43,185,040 (15.5)	42,561,872 (15.9)	623,169 (5.7)
50–59	6707 (15.7)	6406 (15.6)	301 (17.4)	42,679,091 (15.3)	40,629,523 (15.2)	2,049,568 (18.7)
60–74	6678 (15.6)	6165 (15.0)	513 (29.7)	43,420,164 (15.6)	40,300,696 (15.1)	3,119,467 (28.5)
> =75	4873 (11.4)	4141 (10.1)	732 (42.4)	31,346,071 (11.2)	26,674,553 (10.0)	4,671,518 (42.7)
Race/ethnicity						
White	27,251 (63.6)	26,014 (63.3)	1237 (71.7)	175,775,546 (63.1)	168,129,691 (62.8)	7,645,855 (69.9)
Black	9207 (21.5)	8857 (21.5)	350 (20.3)	62,663,628 (22.5)	60,262,819 (22.5)	2,400,808 (21.9)
Hispanic	5152 (12.0)	5038 (12.3)	114 (6.6)	33,391,671 (12.0)	32,679,875 (12.2)	711,796 (6.5)
Asian	804 (1.9)	793 (1.9)	11 (0.6)	4,392,213 (1.6)	4,299,639 (1.6)	92,574 (0.8)
Other	418 (1.0)	404 (1.0)	14 (0.8)	2,475,999 (0.9)	2,385,693 (0.9)	90,306 (0.8)
Residence type						
Private residence	39,819 (95.1)	38,335 (95.3)	1484 (88.3)	258,354,513 (95.3)	248,844,348 (95.5)	9,510,164 (89.1)
Nursing home	885 (2.1)	725 (1.8)	160 (9.5)	5,875,161 (2.2)	4,935,676 (1.9)	939,486 (8.8)
Homeless	534 (1.3)	524 (1.3)	10 (0.6)	2,480,109 (0.9)	2,449,251 (0.9)	30,858 (0.3)
Other	651 (1.6)	624 (1.6)	27 (1.6)	4,501,686 (1.7)	4,309,055 (1.7)	192,631 (1.8)
Insurance type						
Private insurance	12,446 (30.8)	12,274 (31.7)	172 (10.3)	79,443,111 (30.5)	78,392,584 (31.4)	1,050,526 (10.0)
Medicare	10,517 (26.0)	9331 (24.1)	1186 (71.1)	66,956,323 (25.7)	59,480,378 (23.8)	7,475,945 (71.0)
Medicaid or CHIP	11,148 (27.6)	10,914 (28.2)	234 (14.0)	71,529,605 (27.5)	69,979,439 (28.0)	1,550,166 (14.7)
Uninsured	4886 (12.1)	4833 (12.5)	53 (3.2)	33,248,283 (12.8)	32,969,016 (13.2)	279,267 (2.7)
Other	1406 (3.5)	1382 (3.6)	24 (1.4)	9,371,908 (3.6)	9,204,758 (3.7)	167,149 (1.6)
Year						
2014	15,319 (35.8)	14,710 (35.8)	609 (35.3)	90,554,699 (32.5)	87,207,276 (32.6)	3,347,423 (30.6)
2015	14,041 (32.8)	13,456 (32.7)	585 (33.9)	89,005,064 (31.9)	85,124,272 (31.8)	3,880,792 (35.5)
2016	13,472 (31.5)	12,940 (31.5)	532 (30.8)	99,139,294 (35.6)	95,426,169 (35.6)	3,713,125 (33.9)
Day of Week						
Sunday	5622 (13.1)	5423 (13.2)	199 (11.5)	35,918,011 (12.9)	34,596,685 (12.9)	1,321,326 (12.1)
Monday	6930 (16.2)	6641 (16.2)	289 (16.7)	44,958,717 (16.1)	43,076,353 (16.1)	1,882,364 (17.2)
Tuesday	6347 (14.8)	6083 (14.8)	264 (15.3)	40,922,676 (14.7)	39,201,202 (14.6)	1,721,474 (15.7)
Wednesday	6225 (14.5)	5977 (14.5)	248 (14.4)	40,888,226 (14.7)	39,299,657 (14.7)	1588,568 (14.5)
Thursday	5952 (13.9)	5711 (13.9)	241 (14.0)	39,069,043 (14.0)	37,527,610 (14.0)	1,541,433 (14.1)
Friday	5960 (13.9)	5693 (13.8)	267 (15.5)	38,869,467 (13.9)	37,170,488 (13.9)	1,698,979 (15.5)
Saturday	5796 (13.5)	5578 (13.6)	218 (12.6)	38,072,918 (13.7)	36,885,722 (13.8)	1,187,196 (10.9)
Arrive by ambulance	7729 (18.5)	7023 (17.6)	706 (41.8)	49,769,047 (18.3)	45,250,252 (17.3)	4,518,795 (42.1)
Seen within last 72 h	1914 (4.9)	1835 (4.9)	79 (5.0)	11,953,039 (4.8)	11,584,897 (4.8)	368,142 (3.7)
Pain level						
No pain	7711 (24.4)	7216 (23.7)	495 (41.0)	46,478,004 (23.1)	43,617,929 (22.6)	2,860,075 (37.4)
Mild	2916 (9.2)	2827 (9.3)	89 (7.4)	18,235,636 (9.1)	17,613,003 (9.1)	622,633 (8.1)
Moderate	9430 (29.8)	9146 (30.0)	284 (23.5)	60,509,861 (30.1)	58,733,139 (30.4)	1,776,722 (23.2)
Severe	11,602 (36.6)	11,262 (37.0)	340 (28.1)	75,762,102 (37.7)	73,367,325 (37.9)	2,394,777 (31.3)
Temperature						
36 °C–38 °C	38,083 (94.6)	36,621 (94.7)	1462 (92.2)	249,171,894 (95.1)	239,846,217 (95.1)	9,325,676 (93.2)
< =36 °C	1522 (3.8)	1429 (3.7)	93 (5.9)	9,089,224 (3.5)	8,547,754 (3.4)	541,470 (5.4)
> 38 °C	635 (1.6)	605 (1.6)	30 (1.9)	3,863,922 (1.5)	3,723,158 (1.5)	140,763 (1.4)
Heart Rate, times/min						
< =90	28,489 (66.5)	27,286 (66.4)	1203 (69.7)	184,822,552 (66.3)	177,061,313 (66.1)	7,761,239 (70.9)
90–100	7169 (16.7)	6930 (16.9)	239 (13.8)	46,314,663 (16.6)	44,873,977 (16.8)	1,440,686 (13.2)
100–110	3906 (9.1)	3773 (9.2)	133 (7.7)	25,427,295 (9.1)	24,645,034 (9.2)	782,261 (7.1)
110–120	1988 (4.6)	1900 (4.6)	88 (5.1)	13,118,183 (4.7)	12,537,614 (4.7)	580,569 (5.3)
> 120	1280 (3.0)	1217 (3.0)	63 (3.7)	9,016,363 (3.2)	8,639,779 (3.2)	376,583 (3.4)
DBP mm Hg						
60–80	19,358 (45.2)	18,560 (45.2)	798 (46.2)	125,677,278 (45.1)	120,623,428 (45.0)	5,053,850 (46.2)
< 60	4312 (10.1)	3965 (9.6)	347 (20.1)	26,198,088 (9.4)	23,931,635 (8.9)	2,266,453 (20.7)
> 80	19,162 (44.7)	18,581 (45.2)	581 (33.7)	126,823,690 (45.5)	123,202,654 (46.0)	3,621,036 (33.1)
SBP mm Hg						
80–120	9773 (22.8)	9322 (22.7)	451 (26.1)	61,351,488 (22.0)	58,394,721 (21.8)	2,956,767 (27.0)
< 80	1588 (3.7)	1513 (3.7)	75 (4.3)	9,419,022 (3.4)	9,005,031 (3.4)	413,990 (3.8)
> 120	31,471 (73.5)	30,271 (73.6)	1200 (69.5)	207,928,547 (74.6)	200,357,965 (74.8)	7,570,582 (69.2)
Census Region						
Northeast	7176 (16.8)	6937 (16.9)	239 (13.8)	43,967,048 (15.8)	42,383,250 (15.8)	1,583,797 (14.5)
Midwest	10,893 (25.4)	10,343 (25.2)	550 (31.9)	74,304,118 (26.7)	71,017,621 (26.5)	3,286,496 (30.0)
South	15,430 (36.0)	14,760 (35.9)	670 (38.8)	105,760,507 (37.9)	101,247,049 (37.8)	4,513,457 (41.3)
West	9333 (21.8)	9066 (22.1)	267 (15.5)	54,667,385 (19.6)	53,109,796 (19.8)	1,557,589 (14.2)
This visit is related to						
Injury/trauma	12,286 (30.1)	12,006 (30.7)	280 (17.1)	78,178,483 (29.5)	76,464,334 (30.0)	1,714,149 (16.6)
Overdose/poisoning	499 (1.2)	490 (1.3)	9 (0.5)	3,358,380 (1.3)	3,313,963 (1.3)	44,417 (0.4)
Adverse effect of medical/surgical treatment	1099 (2.7)	1038 (2.7)	61 (3.7)	7,170,683 (2.7)	6,768,702 (2.7)	401,982 (3.9)
Visit not related to any above	26,692 (65.4)	25,413 (64.9)	1279 (78.1)	174,903,611 (66.0)	166,770,439 (65.4)	8,133,172 (78.6)
Questionable injury status	214 (0.5)	206 (0.5)	8 (0.5)	1,546,669 (0.6)	1,494,095 (0.6)	52,574 (0.5)

The proportion of ED visits by patients with CHF varied by US census region: Northeast, 14.5%; Midwest, 30.0%; South, 41.3%; and West, 14.2% (*p* < 0.01). A greater proportion of ED patients with CHF belonged to the 50–59, 60–74, and > =75 age groups when compared to their non-CHF counterparts (18.7 vs. 15.2%, 28.5 vs. 15.1%, and 42.7 vs. 10.0%, respectively, *p* < 0.001). ED patients with CHF comprised a higher proportion of non-Hispanic Whites relative to those without CHF (69.9 vs. 62.8%, *p* < 0.001).

Table 2 describes the selected reasons for visit and emergency diagnosis among ED patients with CHF. Compared with their non-CHF counterparts, patients with CHF have a greater proportion of visits to the ED for general symptoms, symptoms referable to the cardiovascular and lymphatic system, and symptoms referable to the respiratory system (26.6% vs. 19.0, 3.4% vs. 2.1, 25.2% vs. 9.4%, respectively).

**Table 2:** Selected Reason for Visit and Emergency Department Diagnosis among ED Patients with Congestive heart failure, NHAMCS 2014–2016

	Unweighted Sample			Weighted Sample		
	All	No Congestive heart failure	Congestive heart failure	All	No Congestive heart failure	Congestive heart failure
Reason for visit						
General Symptoms	8187 (19.1)	7734 (18.8)	453 (26.3)	53,664,580 (19.3)	50,769,980 (19.0)	2,894,600 (26.6)
Symptoms Referable to Psychological and Mental Disorders	1700 (4.0)	1646 (4.0)	54 (3.1)	9,426,523 (3.4)	9,138,510 (3.4)	288,013 (2.6)
Symptoms Referable to the Nervous System	3304 (7.7)	3234 (7.9)	70 (4.1)	20,833,741 (7.5)	20,455,816 (7.7)	377,925 (3.5)
Symptoms Referable to the Cardiovascular and Lymphatic Systems	889 (2.1)	823 (2.0)	66 (3.8)	5,993,917 (2.2)	5,625,049 (2.1)	368,869 (3.4)
Symptoms Referable to the Eyes and Ears	848 (2.0)	839 (2.0)	9 (0.5)	5,778,778 (2.1)	5,721,193 (2.1)	57,585 (0.5)
Symptoms Referable to the Respiratory System	4198 (9.8)	3721 (9.1)	477 (27.7)	27,856,021 (10.0)	25,110,566 (9.4)	2,745,455 (25.2)
Symptoms Referable to the Digestive System	6807 (15.9)	6629 (16.2)	178 (10.3)	46,038,272 (16.5)	44,684,121 (16.7)	1,354,151 (12.4)
Symptoms Referable to the Genitourinary System	2477 (5.8)	2442 (6.0)	35 (2.0)	14,984,361 (5.4)	14,774,942 (5.5)	209,419 (1.9)
Symptoms Referable to the Skin, Nails, and Hair	1333 (3.1)	1303 (3.2)	30 (1.7)	8,716,118 (3.1)	8,534,035 (3.2)	182,083 (1.7)
Symptoms Referable to the Musculoskeletal System	6519 (15.2)	6356 (15.5)	163 (9.5)	42,820,579 (15.4)	41,594,610 (15.6)	1,225,969 (11.2)
Other	6501 (15.2)	6312 (15.4)	189 (11.0)	42,147,135 (15.1)	40,949,503 (15.3)	1,197,632 (11.0)

Supplemental Table 1 describes associations between the patients’ characteristics and their CHF status. Compare to female ED patients, males were 1.35 times more likely to have CHF (aOR: 95% CI: 1.22–1.50). Among ED patients, Black patients were 1.27 times more likely than White patients to have CHF (aOR: 95% CI: 1.11–1.46), while Asian patients were 58% less likely than White patients to have CHF (aOR: 0.42; 95% CI: 0.23–0.77). Compared to ED patients inhabiting a private residence, those who were from nursing homes were 1.24 times more likely to have CHF (aOR: 1.24; 95% CI: 1.01–1.52). Compared to ED patients with private insurance, those with Medicare and Medicaid or CHIP were 2.62 (95% CI: 2.19–3.13) and 1.90 times (95% CI: 1.54–2.34), respectively, more likely to have CHF. Patients who arrived by ambulance were 1.86 times more likely to have CHF (95% CI: 1.66–2.09). Additionally, ED patients who presented with adverse effects of medical/surgical treatment were 1.62 more likely to have CHF than those presenting with injury or trauma (aOR: 1.62; 95% CI: 1.20–2.19).

Tables 3 and 4 describe the proportions of patients by ESI, hospital admission, ICU admission, and medical resources utilization. The hospital admission rate among ED patients was 2.56 times higher for patients with CHF (95% CI: 2.28–2.87); patients have CHF were also 3.03 times more likely to receive immediate vs. semi- or non-urgent ESI scores compared to patients without CHF (95% CI: 2.48–3.71). The intensive care unit admission rate was 2.19 times higher in patients with CHF (95% CI: 1.77–2.71). ED patients with CHF were 2.54 times more likely to receive blood tests (95% CI: 2.21–2.91), and were more likely to utilize other medical resources; for example, they were 1.69 times more likely to get an ultrasound scan compared to patients without CHF (95% CI: 1.33–2.15), and were 1.18 times more likely to receive an ultrasound scan compared to patients without procedure (95% CI: 1.06–1.30).

**Table 3:** Proportion of Emergency Severity Index, Hospital admission, ICU admission, Medical resources utilization, stratified by Congestive heart failure, NHAMCS 2014–2016

	Unweighted Sample			Weighted Sample		
	All	No CHF	CHF	All	No CHF	CHF
ESI score						
Immediate	239 (0.8)	235 (0.8)	4 (1.5)	1,496,327 (0.8)	1,471,879 (0.7)	24,448 (1.7)
Emergent	3615 (11.6)	3529 (11.5)	86 (32.8)	23,433,327 (11.8)	22,976,847 (11.7)	456,480 (31.6)
Urgent	15,392 (49.5)	15,248 (49.5)	144 (55.0)	97,000,149 (49.0)	96,286,096 (49.0)	714,053 (49.4)
Semi-urgent	10,051 (32.3)	10,034 (32.6)	17 (6.5)	65,085,335 (32.9)	64,950,854 (33.0)	134,480 (9.3)
Non-urgent	1784 (5.7)	1773 (5.8)	11 (4.2)	11,046,598 (5.6)	10,931,909 (5.6)	114,689 (7.9)
Hospital Admission	5852 (13.7)	5695 (13.4)	157 (42.8)	36,388,538 (13.1)	35,517,254 (12.8)	871,284 (40.2)
ICU	698 (1.6)	669 (1.6)	29 (7.9)	4,647,353 (1.7)	4,506,861 (1.6)	140,492 (6.5)
In hospital death	201 (0.5)	192 (0.5)	9 (2.5)	1,342,510 (0.5)	1,298,220 (0.5)	44,290 (2.0)
Left before/after triage	1085 (2.5)	1076 (2.5)	9 (2.5)	6,792,175 (2.4)	6,722,799 (2.4)	69,376 (3.2)
Blood test	21,958 (51.3)	21,654 (51.0)	304 (82.8)	142,656,097 (51.2)	140,833,111 (50.9)	1,822,986 (84.1)
Any image	21,950 (51.2)	21,709 (51.1)	241 (65.7)	144,824,612 (52.0)	143,375,099 (51.8)	1,449,513 (66.9)
X-ray	15,099 (35.3)	14,894 (35.1)	205 (55.9)	99,429,274 (35.7)	98,179,495 (35.5)	1,249,778 (57.6)
CT	8414 (19.6)	8338 (19.6)	76 (20.7)	54,986,804 (19.7)	54,559,942 (19.7)	426,863 (19.7)
Ultrasound	2218 (5.2)	2205 (5.2)	13 (3.5)	14,936,538 (5.4)	14,833,060 (5.4)	103,478 (4.8)
MRI	446 (1.0)	438 (1.0)	8 (2.2)	2,831,626 (1.0)	2,791,440 (1.0)	40,186 (1.9)
Other Imaging	604 (1.4)	595 (1.4)	9 (2.5)	4,297,097 (1.5)	4,239,282 (1.5)	57,815 (2.7)
Procedure	21,021 (49.1)	20,807 (49.0)	214 (58.3)	133,801,012 (48.0)	132,620,938 (48.0)	1,180,074 (54.4)
Waiting time (minutes, MEANS (95% CI))	41.1 (40.3–41.8)	41.0 (40.3–41.8)	45.2 (37.7–52.7)	39.9 (39.2–40.6)	39.9 (39.1–40.6)	46.5 (38.8–54.1)
Length of visit (minutes, MEANS (95% CI))	245.6 (241.6–249.6)	244.1 (240.1–248.1)	422.7 (339.2–506.3)	230.2 (226.7–233.8)	228.7 (225.2–232.2)	450.3 (358.0–542.5)

Notes: Waiting time—time from arrival to seeing the physician; Length of visit—time from arrival to discharge

**Table 4:** Odds Ratio of Emergency Severity Index, Hospital admission, ICU admission, Medical Resources Utilization for Congestive heart failure vs. Non-congestive heart failure Patients, NHAMCS 2014–2016

	Crude Odds Ratio	Adjusted for		
		Demographics	+ Social economic	+ Visiting & Clinical
ESI Score: Immediate or Emergent vs. Semi- or Non-Urgent	6.92 (5.77–8.30)	3.98 (3.30–4.81)	3.87 (3.21–4.68)	3.03 (2.48–3.71)
ESI Score: Urgent vs. Semi- or Non-Urgent	3.24 (2.75–3.82)	2.32 (1.96–2.75)	2.28 (1.93–2.70)	2.07 (1.74–2.47)
Hospital Admission	5.78 (5.24–6.39)	3.14 (2.83–3.49)	3.00 (2.70–3.33)	2.56 (2.28–2.87)
ICU	6.85 (5.67–8.28)	3.43 (2.82–4.19)	3.17 (2.59–3.87)	2.19 (1.77–2.71)
Death	5.11 (3.54–7.38)	1.98 (1.36–2.90)	1.87 (1.27–2.74)	1.05 (0.69–1.62)
Left before/after triage	0.46 (0.30–0.72)	0.71 (0.46–1.11)	0.64 (0.41–1.00)	0.60 (0.38–0.93)
Blood test	4.43 (3.92–5.01)	2.93 (2.59–3.33)	2.88 (2.53–3.27)	2.54 (2.21–2.91)
Any imaging	2.91 (2.61–3.25)	1.86 (1.66–2.09)	1.85 (1.65–2.07)	1.77 (1.57–1.99)
X-ray	3.52 (3.18–3.89)	2.27 (2.04–2.52)	2.21 (1.99–2.45)	2.00 (1.79–2.24)
CT	1.33 (1.19–1.49)	0.83 (0.74–0.93)	0.82 (0.73–0.93)	0.90 (0.79–1.02)
Ultrasound	0.96 (0.77–1.20)	1.43 (1.13–1.80)	1.52 (1.20–1.92)	1.69 (1.33–2.15)
MRI	0.83 (0.49–1.39)	0.54 (0.32–0.91)	0.59 (0.35–1.00)	0.76 (0.44–1.30)
Procedure	1.33 (1.21–1.46)	1.19 (1.08–1.31)	1.19 (1.08–1.32)	1.18 (1.06–1.30)

Note: ^*^ + Demographics include: gender, age group, race/ethnicity; +Social economic: residence type, insurance type, census region; + Visiting & Clinical: year, week of day, arrive by ambulance, seen within last 72 h, pain level, temperature, heart rate, dialytic blood pressure, injury status, reason for visit

## Discussion

We presented a comprehensive study on the national characteristics of ED patients with CHF history [14, 23]. Past studies were focused on regionally or only included the ED samples of patients with CHF [14, 23–25]. The current study described the ED patients with CHF with greater power by analyzing a larger, more representative sample. Emergency department is an important component of the CHF healthcare delivery system. Better understanding of the clinical characteristics and health outcomes of CHF ED patients is a first step to improving the healthcare and treatment of these patients as well as ED encounters in this population.

This study reports new epidemiological characteristics on ED patients with CHF including their demographics, presenting vitals, hospital/ICU admission, and medical imaging / lab test resource utilization. We found demographic differences were associated with the prevalence of CHF in ED patients. For example, EDs in the South region had the largest proportion of visits by patients with CHF. Female ED patients were less likely than males to have CHF, and non-Hispanic White patients were more likely to have CHF than other races/ethnicities. These differences are generally consistent with the demographic patterns in CHF prevalence in the US. We also found that CHF patients with Medicare and Medicaid or CHIP were more likely to visit the ED than were patients with private insurance. This higher likelihood of ED visitation in CHF patients with Medicare and Medicate or CHIP could be related to increased medication nonadherence in CHF patients with Medicare and Medicaid [26]. CHF patients who visited the ED were also more likely to arrive by ambulance likely because patients with worsening CHF may be more likely to use an ambulance than other types of patients [27]. These findings indicate that increased focus on medication adherence in Medicare and Medicaid patients as well as further attention to management of CHF patients during ambulance transfer may be pertinent areas for further research to improve clinical outcomes.

Patients with CHF had over 3.6 million ED visits each year nationally. Compared to non-CHF ED visits, patients with CHF in the ED present higher ESI scores, higher chance of hospital/ICU admission, as well as undergoing a blood test and ultrasound scan. This indicates that a higher level of emergency care is needed among patients with CHF require. This increased use of hospital resources is consistent with previous research suggesting greater utilization of hospital resources by CHF patients, including a significant rate of hospital readmission among CHF patients [28]. This indicates that better allocation of ED, and intensive care units with resources for preparedness and better case management of elderly black or non-Hispanic White are needed for patients with CHF.

Exploration of the reasons for ED visits and revisits among CHF patients may contribute to the development of interventions to reduce ED visits and revisit rates among these patients. Emergency department is an understudied setting for CHF healthcare delivery, considering the large number of ED visits among patients with CHF. More detailed patients’ history data of CHF are needed for future study, which can help inform approaches of increasing these patients’ use of routine care over ED care. The higher rate of hospital and ICU admission indicates that CHF patients require a higher level of emergency care. These findings suggest that increased recognition of the potential of the ED as a high-leverage setting for improving treatment and screening of CHF is needed, which can be developed by identifying characteristics and trajectories of patients presenting to the ED with CHF.

There were several limitations to the present study. First, information regarding to the severity and stage of CHF was not gathered in NHAMCS-ED dataset. Secondly, the NHAMCS-ED survey data does not contains information on treatment history (e.g. pharmacologic) for CHF. Future studies may examine more data related to CHF characteristics, comorbidities, treatment history, and other finer-grained clinical data, which allows for better understanding of the associated factors related to CHF ED care. Thirdly, the data on the acute and chronic CHF is unknown in the survey, which should be collected in the future studies.

## Conclusion

This study describes the demographic, socioeconomic, and clinical characteristics of patients who present to the ED with CHF through analysis of a comprehensive national survey. The study describes the characteristics of CHF patients who visit the ED on a national scale. We found that there are gender, age, and racial/ethnic differences between ED patients with and without CHF. These patients require a higher level of emergency care due to their higher chance of admittance to the hospital and ICU.

## Supplementary Information

**Additional file 1: Supplement Table 1.** Association between ED visiting with Congestive heart failure and patient visiting characteristics, NHAMCS 2014–2016. Note: the adjusted OR was from a logistic regression including all variables in the table.

## Data Availability

The NHAMCS-ED dataset can be accessed through the website of the US Centers for Disease Control and Prevention (CDC) (https://www.cdc.gov/nchs/ahcd/index.htm). The detailed explanation of the survey data for each year and the code book can be found here: https://ftp.cdc.gov/pub/Health_ Statistics/NCHS/dataset_documentation/nhamcs/.

## Abbreviations

aOR: Adjusted odds ratio
CI: Confidential interval
ED: Emergency department
NHAMCS-ED: National Hospital Ambulatory Medical Care Survey ED Subfile
ESI: Emergency severity index
ICU: Intensive care unit
CT: Computed tomography
MRI: Magnetic resonance imaging
CHF: Congestive heart failure
