# Supplementary material for: Characteristics of emergency department admissions with congestive heart failure in the United States: a Nationwide cross-sectional study
Source: BMC Emerg Med. 2022 Jan 28;22:16. doi: 10.1186/s12873-021-00564-7 (PMC8795967; doi:10.1186/s12873-021-00564-7)
Supplement: Supplementary file 1 — Additional file 1: Supplement Table 1. Association between ED visiting with Congestive heart failure and patient visiting characteristics, NHAMCS 2014–2016. Note: the adjusted OR was from a logistic regression including all variables in the table. [file 12873_2021_564_MOESM1_ESM.docx]

Supplement Table 1. Association between ED visiting with Congestive heart failure and patient visiting characteristics, NHAMCS 2014–2016

| Effect | Crude OR (95% CI) | Adjusted OR (95% CI) |
| --- | --- | --- |
| Age |  |  |
| 18–39 | Reference [1] | Reference [1] |
| 40–49 | 3.64(2.70-4.89) | 3.42(2.54-4.62) |
| 50–59 | 10.88(8.46-14.00) | 9.02(6.97-11.67) |
| 60–74 | 19.27(15.14-24.53) | 11.05(8.53-14.32) |
| >=75 | 40.94(32.30-51.90) | 18.63(14.26-24.34) |
| Male vs Female | 1.30(1.18-1.43) | 1.35(1.22-1.50) |
| Race/ethnicity |  |  |
| White | Reference [1] | Reference [1] |
| Black | 0.83(0.74-0.94) | 1.27(1.11-1.46) |
| Hispanic | 0.48(0.39-0.58) | 0.85(0.69-1.05) |
| Asian | 0.29(0.16-0.53) | 0.42(0.23-0.77) |
| Other | 0.73(0.43-1.25) | 1.28(0.73-2.24) |
| Day of Week |  |  |
| Sunday | Reference [1] | Reference [1] |
| Monday | 1.19(0.99-1.43) | 1.17(0.96-1.42) |
| Tuesday | 1.18(0.98-1.43) | 1.17(0.96-1.43) |
| Wednesday | 1.13(0.94-1.37) | 1.14(0.93-1.40) |
| Thursday | 1.15(0.95-1.39) | 1.15(0.93-1.41) |
| Friday | 1.28(1.06-1.54) | 1.25(1.03-1.53) |
| Saturday | 1.07(0.88-1.30) | 1.08(0.88-1.33) |
| Year |  |  |
| 2014 | Reference [1] | Reference [1] |
| 2015 | 1.05(0.94-1.18) | 1.04(0.92-1.18) |
| 2016 | 0.99(0.88-1.12) | 0.98(0.87-1.12) |
| Residence type |  |  |
| Private residence | Reference [1] | Reference [1] |
| Nursing home | 5.66(4.74-6.77) | 1.24(1.01-1.52) |
| Homeless | 0.49(0.26-0.92) | 0.59(0.31-1.12) |
| Other | 1.11(0.75-1.64) | 0.65(0.43-0.98) |
| Insurance type |  |  |
| Private insurance | Reference [1] | Reference [1] |
| Medicare | 7.58(6.45-8.91) | 2.62(2.19-3.13) |
| Medicaid or CHIP | 1.53(1.25-1.87) | 1.90(1.54-2.34) |
| Uninsured | 0.78(0.57-1.07) | 1.17(0.85-1.61) |
| Other | 1.24(0.81-1.91) | 1.35(0.87-2.11) |
| Temperature |  |  |
| 36 °C–38 °C | Reference [1] | Reference [1] |
| <=36 °C | 1.59(1.28-1.97) | 1.26(1.00-1.59) |
| >38 °C | 1.21(0.84-1.75) | 0.70(0.47-1.04) |
| Heart Rate |  |  |
| <=90 | Reference [1] | Reference [1] |
| 90–100 | 0.78(0.68-0.90) | 0.98(0.84-1.14) |
| 100–110 | 0.80(0.67-0.96) | 0.99(0.81-1.20) |
| 110–120 | 1.05(0.84-1.31) | 1.41(1.10-1.80) |
| >120 | 1.17(0.91-1.52) | 1.08(0.81-1.44) |
| DBP |  |  |
| 60–80 | Reference [1] | Reference [1] |
| <60 | 2.04(1.79-2.32) | 1.47(1.28-1.70) |
| >80 | 0.73(0.65-0.81) | 0.79(0.70-0.89) |
| Pain level |  |  |
| No pain | Reference [1] | Reference [1] |
| Mild | 0.46(0.37-0.58) | 0.85(0.66-1.08) |
| Moderate | 0.59(0.53-0.66) | 1.01(0.89-1.15) |
| Severe | 0.44(0.38-0.51) | 1.12(0.95-1.31) |
| 72 hours revisit vs not | 0.97(0.77-1.23) | 0.91(0.71-1.17) |
| Ambulance arrival vs not | 3.36(3.04-3.71) | 1.86(1.66-2.09) |
| Census Region |  |  |
| Northeast | Reference [1] | Reference [1] |
| Midwest | 1.54(1.32-1.80) | 1.47(1.24-1.73) |
| South | 1.32(1.13-1.53) | 1.33(1.14-1.57) |
| West | 0.86(0.72-1.02) | 0.96(0.80-1.16) |
| Reason for visit |  |  |
| General Symptoms | Reference [1] | Reference [1] |
| Symptoms Referable to Psychological and Mental Disorders | 0.56(0.42-0.75) | 0.56(0.41-0.76) |
| Symptoms Referable to the Nervous System | 0.37(0.29-0.48) | 0.42(0.32-0.54) |
| Symptoms Referable to the Cardiovascular and Lymphatic Systems | 1.37(1.05-1.79) | 0.96(0.73-1.28) |
| Symptoms Referable to the Eyes and Ears | 0.18(0.09-0.36) | 0.38(0.19-0.74) |
| Symptoms Referable to the Respiratory System | 2.19(1.91-2.50) | 2.16(1.87-2.51) |
| Symptoms Referable to the Digestive System | 0.46(0.38-0.55) | 0.62(0.51-0.74) |
| Symptoms Referable to the Genitourinary System | 0.25(0.17-0.35) | 0.49(0.34-0.70) |
| Symptoms Referable to the Skin, Nails, and Hair | 0.39(0.27-0.57) | 0.73(0.49-1.07) |
| Symptoms Referable to the Musculoskeletal System | 0.44(0.37-0.53) | 0.64(0.53-0.78) |
| Other | 0.51(0.43-0.61) | 0.76(0.62-0.93) |
| Is this visit related to |  |  |
| Injury/trauma | Reference [1] | Reference [1] |
| Overdose/poisoning | 0.79(0.40-1.54) | 0.90(0.45-1.79) |
| Adverse effect of medical/surgical treatment | 2.52(1.90-3.35) | 1.62(1.20-2.19) |
| Visit not related to any above | 2.14(1.88-2.44) | 1.81(1.53-2.13) |
| Questionable injury status | 1.67(0.81-3.41) | 1.54(0.72-3.30) |

Note: the adjusted OR was from a logistic regression including all variables in the table.
